# Advances in Development of Countermeasures for Potential Biothreat Agents

**DOI:** 10.1155/2012/570246

**Published:** 2012-07-16

**Authors:** Phillip R. Pittman, Kelly T. McKee, Zygmunt F. Dembek

**Affiliations:** ^1^Department of Clinical Research, United States Army Medical Research Institute of Infectious Diseases (USAMRIID), Fort Detrick, Frederick, MD 21704-5011, USA; ^2^Public Health and Government Services, Quintiles Transnational Corporation, Durham, NC 27703, USA; ^3^Center for Disaster and Humanitarian Assistance Medicine, Uniformed Services University of The Health Sciences, Bethesda, MD 20814-4779, USA

September 11, 2001, was the day that the people of the United States of America came face to face with the specter of terrorism. Long considered secure by virtue of being bordered by friendly countries to the north and south and two great bodies of water to the east and west, the US landmass was suddenly no longer safe from the brutal and indiscriminate acts of extreme mass violence that had menaced other countries for decades. A few weeks later, and already shaken to its core, the anthrax spore letter attacks then awakened the American public to the reality of lethal biological terrorism. The autumn of 2001 was, indeed, a frightening and unsettling time.

Before this, the potential for use of live biological agents and toxins as weapons was viewed largely to be the purview of the US military; revelations of the large-scale Soviet program and discovery of weapons caches and warheads in Iraq after the first Gulf War had raised general awareness of the threat, but had done little to stimulate the political will necessary to invest in medical countermeasures before 2001. The anthrax letter attacks, however, served to crystallize the threat, and, for the first time, significant funds were allocated by the US Congress for biodefense. 

The original goal of this special edition of Advances in Preventive Medicine was to provide a venue for highlighting some of the advances in biodefense research which have been made since the fall of 2001. Papers selected by the editors for inclusion include an overview of biosurveillance; a consideration of the regulatory hurdles needed to be overcome for FDA licensure of potentially beneficial products; a discussion of correlates of immunity; and two papers highlighting progress in the development of new vaccines to counter biothreat agents. 

A reliable surveillance program is a key component of the early detection algorithms necessary to institute countermeasures in a timely manner. The paper by Kman and Bachmann, “Biosurveillance: a review and update,” offers a discussion of passive and active surveillance, and provides overviews of syndromic surveillance, laboratory surveillance, and environment surveillance, together with the systems used in these methodologies and their relative effectiveness. 

The very nature of biologic agents used to incite terror proscribes traditional studies to assess efficacy of medical countermeasures against these agents in human volunteers. The paper by Aebersold, “FDA experience with medical countermeasures under the Animal Rule,” offers a comprehensive review of the development of the regulatory framework established by the US Food and Drug Administration for licensing medical countermeasures against chemical, biological, and radiological threats, the so-called “Animal Rule,” as well as experience in implementing this guidance in the approval of two drugs: pyridostigmine bromide, a drug licensed for the treatment of myasthenia gravis since 1955 and approved by FDA for prophylaxis against the lethal effects of Soman nerve agent poisoning, and Cyanokit, approved as an antidote for patients with known or suspected cyanide poisoning. 

Critical to successful application of the Animal Rule is establishing correlates of protection that can be used to establish surrogate markers of efficacy in humans. The paper by Williamson, “The role of immune correlates and surrogate markers in the development of vaccines and immunotherapies for plague,” provides insight into some of the challenges inherent in identifying such correlates, and approaches to evaluate and validate markers to achieve this objective.

 In “Advanced development of rF1V and rBV A/B vaccines: progress and challenges,” Hart et al. review their experience as Prime Systems contractor for the US Department of Defense in manufacturing, assay development, preclinical and clinical development and testing of vaccines for *Yersinia pestis*, plague, and *Clostridium botulinum* toxins A and B. The authors address obstacles inherent in navigating the requirements of the FDA Animal Rule as they seek to provide safe and effective products that meet the challenges of the modern warfighter. Ricin toxin has remained a menace as an agent of terror. In their paper, “The need for continued development of ricin countermeasures,” the authors, Reisler and Smith, review the origin of the toxin, its pathophysiology, the history of the use of this category B biothreat agent, and the development of medical countermeasures including vaccines. 

This brief collection of papers provides a taste of the challenges underlying development of countermeasures against biological weapons, as well as a sense for the progress that has been made since the early years of the 21st century. It is an unfortunate reality that biological agents are, and will continue to be, threats to public welfare and safety. Continued progress along the lines of the experiences shared here is vital to counter these threats, to remain a step ahead of those who would purposefully harm others through deployment of biological weapons, to prevent illness and death through stimulation of protective immunity, and to mitigate the consequences of their use among the vulnerable.

